# The Contribution of Ultrasound and Doppler Studies on Impaired Intrauterine Conditions and the Development of Future Disease

**DOI:** 10.3390/medicina62050875

**Published:** 2026-05-03

**Authors:** Yossi Geron, Yinon Gilboa, Asaf Romano, Jacob Bar

**Affiliations:** 1 Helen Schneider Hospital for Women, Beilinson Hospital, Rabin Medical Center, Petach Tikva 4941329, Israel; yosefge@clalit.org.il (Y.G.); yinongi@clalit.org.il (Y.G.); asafro@clalit.org.il (A.R.); 2 Gray School of Medicine, Faculty of Medical and Health Sciences, Tel Aviv University, Tel-Aviv 69978, Israel; 3 Obstetrics and Gynecology Department, Wolfson Medical Center, Holon 58100, Israel

**Keywords:** ultrasound, Doppler, fetal growth restriction, overgrowth, large for gestational age, macrosomia, diabetes in pregnancy

## Abstract

The Barker hypothesis links intrauterine conditions, mainly low birth weight, subject to poor nutrition with paradoxically improved standards of living and nutrition after World War II in Western countries, to adult disease, mainly coronary heart disease. The limitations of his hypothesis include the fact that it is based only on human epidemiological data and animal studies, and also that it is difficult to isolate the effect of the intrauterine environment from postnatal conditions, familial and genetic background. In the last 20 years, the introduction of ultrasound and Doppler techniques in the assessment of fetal and maternal vascularity added a major contribution to the evaluation of the intrauterine environment. Studies based on ultrasound and Doppler assist in differentiating between prematurity and fetal growth restriction (FGR), mainly in those with placental insufficiency, and postnatal morbidity and even mortality. In addition, the Pedersen hypothesis regarding fetuses with overgrowth, mainly with diabetic mothers, states that they are also prone to postnatal morbidity. However, most of the studies on the issue do not emphasize the effects of the intrauterine environment on fetal organs, such as the brain, heart, liver, kidneys and pancreas in FGR and fetal overgrowth, that may impose a different prognosis in later life. This narrative review aims to summarize current evidence from animal and human studies regarding the impact of intrauterine undernutrition and overnutrition on fetal organ development, and to evaluate how ultrasound and Doppler findings may contribute to understanding the link between the intrauterine environment and postnatal morbidity.

## 1. Introduction

### The Barker and Pedersen Hypotheses

More than three decades ago, based on findings from human epidemiological and animal studies, the Barker hypothesis was introduced into both the scientific and broader literature [1]. Barker proposed that low birth weight (LBW), subject to poor nutrition in the intrauterine environment with paradoxically improved standards of living and nutrition after World War II in Western countries, results in vascular disease later in life, mainly coronary heart disease. Today, it is well known that intrauterine malnutrition and overnutrition contribute to the metabolic syndrome that includes obesity, impaired glucose tolerance that leads to diabetes mellitus type II, essential hypertension, cerebral ischemia, atherosclerosis, and eventually end-organ damage, mainly to the heart (ischemic heart disease) and kidneys (renal insufficiency). The Barker hypothesis primarily predicts adult disease; however, accumulating evidence suggests that adverse intrauterine conditions may also have consequences during childhood and adolescence. This concept led to the extension of the Barker hypothesis, known as the thrifty phenotype [2], in which fetal adaptation to undernutrition results in fetal growth restriction (FGR) and long-lasting “programmed” metabolic and endocrine alterations. These changes may increase susceptibility later in life to obesity and impaired glucose tolerance (IGT), particularly when postnatal nutrition is abundant, potentially through alterations in cell distribution, gene expression, or both.

In response to reduced placental perfusion and in order to preserve adequate cerebral blood supply, the fetus decreases resistance to blood flow in the middle cerebral artery (MCA). At the same time, arterial redistribution occurs, with increased vascular resistance to other fetal organs, including the heart, kidneys, liver, and pancreas. As a result, exposure to intrauterine ischemia in these organs may lead to long-term consequences such as dyslipidemia, a reduced nephron number, and impaired glucose tolerance, all contributing to metabolic syndrome and end-organ damage later in life. However, support for this hypothesis remains limited and is derived mainly from animal models [3] and retrospective epidemiological studies [4]. Moreover, the hypothesis carries difficulty in isolating the effect of the intrauterine environment from postnatal conditions, family and genetic background. By correlating LBW to later life disease it is difficult to isolate FGR from prematurity that holds a great deal of contribution to morbidity in postnatal life, mainly to the brain (cerebral palsy and developmental disorders) and to the lungs (bronchopulmonary dysplasia and chronic lung disease) [5].

In addition to the Barker hypothesis that linked the intrauterine environment to future health and disease, other investigators suggest a different pathway that links intrauterine life and future disease. The Pedersen hypothesis states that maternal hyperglycemia in a pregnant woman with diabetes stimulates excessive insulin secretion, which is basically a growth hormone, that leads to excessive fetal growth, a state of intrauterine overnutrition. It may happen especially in poorly controlled women with gestational and pre-gestational diabetes. First, these women are prone to developing future metabolic syndrome and end-organ damage [6]. In addition, those with a poorly controlled diabetic pregnancy and to a lesser extent, those with excessive weight gain during their pregnancy [7], by uncontrolled glucose transfer across the placenta, may expose the fetus to overgrowth. Recently, our group demonstrated that even excessive weight gain during pregnancy without diabetes is associated with increased maternal carotid intima-media thickness, adverse pregnancy outcomes (fetal overgrowth), and placental vascular lesions [8]. In more advanced diabetes with a vascular component, early FGR may also be developed [9]. Those offspring exposed to intrauterine overnutrition or undernutrition may develop obesity, IGT, and features of metabolic syndrome, even at a young age [10,11].

Although intrauterine undernutrition and overnutrition represent distinct and often opposite physiological conditions, both can be viewed within the same conceptual framework of developmental programming. In both settings, the fetus adapts to an altered intrauterine environment through organ-specific structural and functional changes, which may have long-term consequences for metabolic and cardiovascular health. This shared adaptive response provides the rationale for addressing these conditions together, despite their differing pathophysiological pathways.

Advances in prenatal imaging, particularly ultrasound and Doppler, now provide an in vivo window into selected fetal and placental manifestations of these adaptive processes.

This review aims to summarize the theoretical background of intrauterine programming, followed by evidence from animal and human studies on fetal organ development in conditions of growth restriction and overgrowth, and finally to describe the role of ultrasound and Doppler in identifying organ-specific fetal adaptations that may serve as early markers of postnatal morbidity.

## 2. Methods

This narrative review was based on a structured literature search conducted in PubMed, MEDLINE, and the Cochrane Library from database inception to March 2026. Search terms included combinations of “intrauterine environment”, “intrauterine programming”, “fetal growth restriction”, “fetal overgrowth”, “large for gestational age (LGA)”, “placental function”, “diabetes in pregnancy”, “ultrasound”, and “Doppler”. Additional relevant studies were identified through manual screening of reference lists of selected articles.

Eligible studies included human and animal studies evaluating structural and functional fetal adaptations in conditions of intrauterine undernutrition and overnutrition, as well as studies assessing ultrasound and Doppler findings in relation to these conditions and their association with postnatal and long-term outcomes. We included original research articles, cohort studies, case series, and key review papers addressing fetal organ development, placental function, or their association with postnatal metabolic and cardiovascular outcomes. Studies not directly relevant to fetal programming or lacking sufficient methodological detail were not emphasized in the synthesis.

Studies were screened based on title and abstract, followed by full-text review for relevance. Screening, selection, and data extraction were performed by two reviewers (Y.Ge. and J.B.).

Data extraction focused on study characteristics, population type (human or animal), intrauterine condition (undernutrition or overnutrition), assessed fetal organ systems, imaging findings, and reported outcomes.

Priority was given to well-designed cohort studies, large observational studies, and studies providing mechanistic insight, including relevant animal models. Landmark studies were included where appropriate to support key concepts.

This review was conducted as a structured narrative review; therefore, no formal systematic review framework (e.g., PRISMA) or quantitative synthesis was applied. Instead, emphasis was placed on a comprehensive and concept-driven synthesis of the available literature.

## 3. Fetal Growth Restriction and Future Health

### 3.1. Animal Studies

Animal studies have provided important mechanistic evidence supporting the concept of intrauterine programming, demonstrating that FGR leads to persistent structural and functional alterations in multiple organ systems. In rat models of placental insufficiency-induced FGR, offspring exhibit reduced brain weight and volume, cortical thinning, abnormal myelination, increased neuroinflammation, and persistent motor and behavioral deficits into adulthood, indicating that the neurological consequences of FGR represent a lasting vulnerability rather than an acute perinatal event [12]. Rabbit models similarly demonstrate that FGR results in impaired pulmonary function, structural lung abnormalities, anxiety-like behavior, impaired memory and attention, and reduced oligodendrocyte numbers in the brain, with these sequelae persisting into preadolescence [13,14].

Piglet models, which spontaneously develop FGR, display brain structural and functional changes analogous to those observed in humans, including neurodevelopmental disabilities such as learning and behavioral disorders, autism, and cerebral palsy [15]. Furthermore, rat studies indicate that FGR followed by early catch-up growth aggravates glucose intolerance, dyslipidemia, and pancreatic islet fibrosis, suggesting an increased risk for metabolic syndrome and type 2 diabetes in adulthood [16,17]. This is consistent with the thrifty phenotype hypothesis, in which fetal adaptation to placental insufficiency results in permanent metabolic and endocrine reprogramming that becomes maladaptive when postnatal nutrition improves.

Collectively, these animal data provide mechanistic support that FGR induces long-term alterations in organ structure and function, particularly affecting the brain, lungs, and metabolic systems.

### 3.2. Human Studies on Fetal Organs: The Brain, Heart, Pancreas, Liver and Kidneys

Evidence from epidemiological and longitudinal human studies indicates that FGR affects multiple organ systems, with structural and functional alterations that originate in utero and extend beyond the perinatal period into childhood, adolescence, and adult life.

#### 3.2.1. Brain

FGR is associated with significant alterations in brain development that begin in utero and persist into postnatal life, despite compensatory hemodynamic adaptations such as the brain-sparing effect. These alterations include reduced total brain volume, preferential involvement of gray matter and cortical structures, as well as reduced cerebellar and hippocampal volumes and abnormalities in white matter organization, indicating region-specific vulnerability of the developing brain [18,19,20].

Reduced cortical folding and gyrification are observed on prenatal MRI, particularly in fetuses with brain-sparing physiology, suggesting that these adaptive circulatory changes may reflect more severe underlying hypoxemia rather than protection [21].

Advanced imaging further demonstrates microstructural and metabolic abnormalities, including reduced apparent diffusion coefficient values across multiple brain regions and decreased N-acetylaspartate ratios, consistent with impaired maturation and neuronal dysfunction [18,22].

At the cellular level, FGR is characterized by impaired oligodendrocyte maturation, delayed myelination, disrupted neuronal development, and increased neuroinflammation, all of which contribute to altered brain organization and connectivity [23,24].

These structural and microstructural abnormalities translate clinically into an increased risk of cognitive delay, learning disabilities, motor dysfunction, and neuropsychological impairments during childhood and adolescence, with cognitive deficits reported in up to 20–40% of affected children [19,25,26,27]. Abnormal Doppler findings, particularly an abnormal cerebroplacental ratio, have been associated with poorer neurodevelopmental outcomes in early childhood [28].

Emerging evidence suggests that the cerebellum may be a region of particular vulnerability in FGR. Reduced cerebellar volume has been associated with adverse neurodevelopmental outcomes, with fetal MRI studies demonstrating correlations between cerebellar size and later cognitive and social function, highlighting the role of the cerebellum beyond motor control [29,30]. Cerebellar hypoplasia in pediatric populations is frequently accompanied by developmental delay, impaired coordination, and autistic features, supporting its role in cognitive and behavioral regulation [31]. Furthermore, studies in children with autism spectrum disorder have identified associations between early cerebellar hypoplasia and later cognitive impairment, as well as altered learning patterns linked to cerebrocerebellar circuitry [32].

Population-based studies have demonstrated a modestly increased risk of autism spectrum disorder among individuals born SGA, supporting an epidemiological association between impaired fetal growth and later neurodevelopmental disorders [33]. While a direct causal relationship between FGR-related cerebellar alterations and autism has not been definitively established, these observations support the hypothesis that disrupted cerebellar development may contribute to increased susceptibility to neurodevelopmental disorders in individuals with impaired fetal growth.

Furthermore, increasing evidence suggests that FGR compromises brain reserve, thereby increasing susceptibility to neurodegenerative and psychiatric disorders later in life, highlighting the long-term impact of altered neurodevelopmental trajectories established during fetal life [34].

#### 3.2.2. Heart and Vascular System

FGR is also associated with significant cardiovascular remodeling that begins in fetal life and persists into postnatal development. A meta-analysis of echocardiographic studies has shown that neonates with FGR exhibit reduced left ventricular mass, decreased interventricular septal thickness, and impaired diastolic function compared with controls [35]. Human studies have demonstrated subclinical myocardial dysfunction, arterial remodeling, and impaired endothelial function in individuals affected by FGR during childhood and adolescence preceding clinically overt cardiovascular disease [36].

Evidence from twin studies further supports a direct effect of the intrauterine environment, demonstrating that the growth-restricted twin exhibits persistent myocardial dysfunction and an increased risk of cardiovascular morbidity independent of genetic factors [37,38]. The long-term cardiovascular consequences of FGR extend into adulthood, with epidemiological studies consistently linking low birth weight and FGR to increased risks of hypertension, coronary artery disease, and stroke, supporting the concept of fetal cardiovascular programming as a contributor to adult cardiovascular morbidity [39].

#### 3.2.3. Pancreas

FGR is consistently associated with long-term metabolic dysfunction, including impaired insulin sensitivity, glucose intolerance, and increased risk of type 2 diabetes mellitus. Human epidemiological and cohort studies demonstrate that low birth weight is associated with adverse metabolic profiles and increased incidence of type 2 diabetes in adulthood, independent of later life risk factors [40,41,42].

Evidence from longitudinal pediatric cohorts suggests that individuals born small for gestational age may exhibit early alterations in glucose metabolism, including impaired insulin secretion despite relatively preserved insulin sensitivity, indicating early β-cell dysfunction [43]. Similarly, studies in growth-restricted neonates have demonstrated reduced pancreatic exocrine activity, suggesting broader impairment of pancreatic development in early life [44].

Adaptive changes in metabolic function are associated with increased metabolic risk later in life, particularly in individuals exposed to rapid postnatal catch-up growth. Consistent with this, altered fetal growth trajectories have been associated with increased insulin resistance in adulthood [45].

#### 3.2.4. Liver

FGR is associated with an increased risk of long-term hepatic dysfunction, particularly non-alcoholic fatty liver disease (NAFLD). Population-based cohort studies demonstrate an association between birth weight and NAFLD in adulthood, supporting a developmental origin of liver disease [46]. Mendelian randomization analyses further suggest a direct causal relationship between lower birthweight and NAFLD risk, independent of maternal factors [47,48].

These effects appear to be mediated by persistent alterations in metabolic pathways, including insulin resistance and lipid metabolism, which promote hepatic steatosis and disease progression [47]. These findings support the concept that FGR induces long-term metabolic programming of the liver, increasing susceptibility to NAFLD later in life.

#### 3.2.5. Kidneys

FGR has been associated with a reduced nephron number and altered renal development, providing a structural basis for long-term renal and cardiovascular disease. These changes are thought to result from intrauterine adaptation to reduced perfusion, leading to permanent alterations in renal architecture and function [49]. Clinically, this manifests as an increased risk of hypertension and chronic kidney disease in later life, supporting the concept of fetal renal programming as a contributor to adult morbidity [50,51].

**Figure 1:**
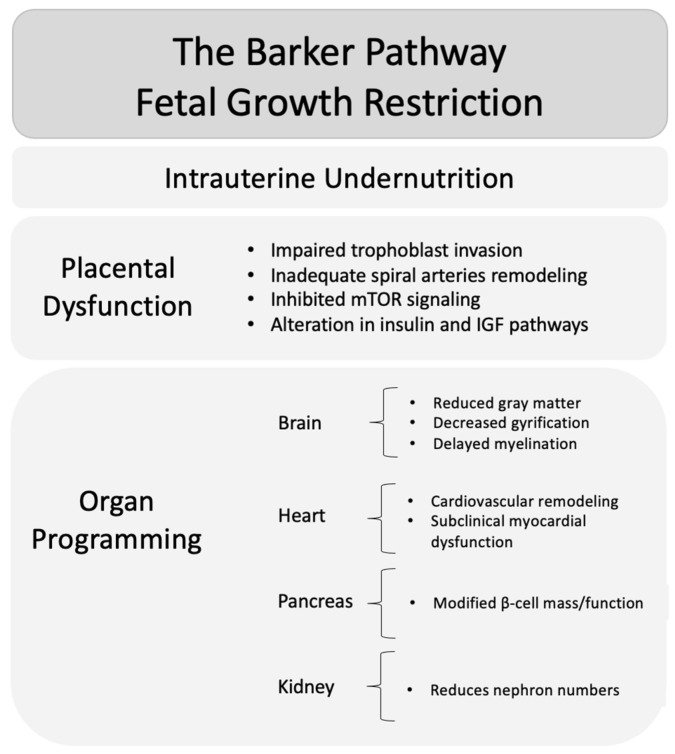
The Barker pathway in fetal growth restriction. Intrauterine undernutrition induces placental dysfunction, leading to fetal growth restriction and organ-specific programming with potential long-term metabolic and cardiovascular consequences.

Collectively, these findings suggest that FGR induces organ-specific structural and functional adaptations that originate in utero and persist throughout life, providing a possible mechanistic link between impaired intrauterine conditions and postnatal morbidity (Figure 1). Despite the consistency of these associations across studies, most data are derived from observational cohorts, and the relative contribution of intrauterine factors compared to genetic and postnatal influences remains difficult to quantify.

**Figure 2:**
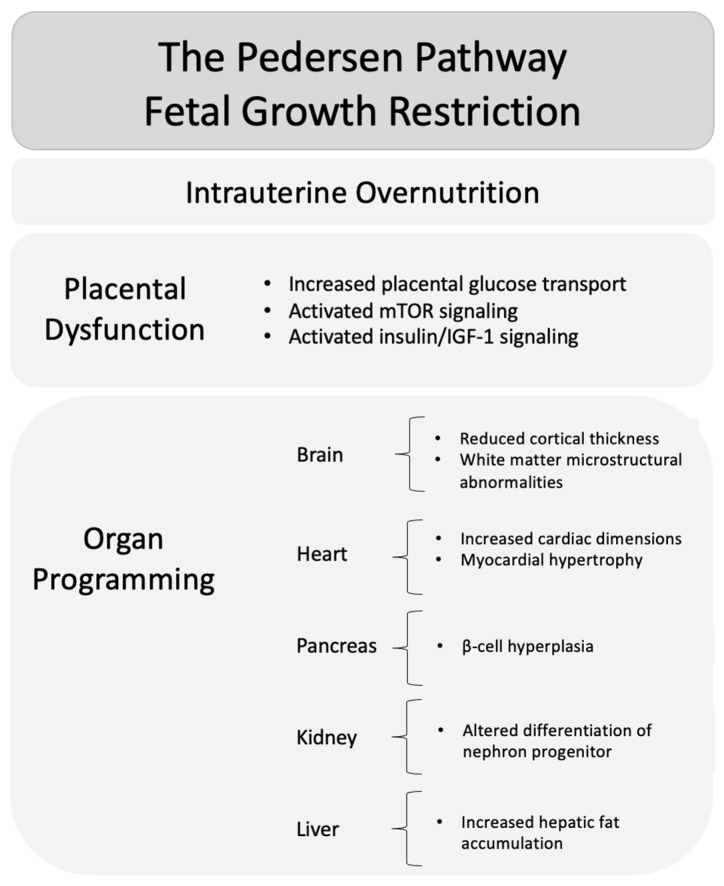
The Pedersen pathway in fetal overgrowth. Intrauterine overnutrition leads to placental metabolic activation, resulting in fetal overgrowth and organ-specific programming with potential long-term metabolic and cardiovascular consequences.

While FGR represents adaptation to intrauterine undernutrition, an opposite but related pathway is observed in conditions of intrauterine overnutrition, characterized by fetal overgrowth (Figure 2).

## 4. Fetal Overgrowth and Future Health

### 4.1. Animal Studies

Animal models have provided important mechanistic insight into the long-term consequences of intrauterine overnutrition and fetal overgrowth. In contrast to fetal growth restriction models, fetal overgrowth in experimental settings is typically induced by maternal diabetes, obesity, or high-fat diet exposure during pregnancy. These conditions increase placental nutrient transfer and stimulate fetal hyperinsulinemia, resulting in fetal overgrowth and metabolic alterations in the offspring.

Rodent models of maternal obesity and high-fat diet have consistently demonstrated that exposure to an obesogenic intrauterine environment predisposes offspring to increased adiposity, insulin resistance, dyslipidemia, and elevated blood pressure later in life [52]. Similarly, experimental models of diabetic pregnancy in rodents produce overgrowth in fetuses and persistent metabolic abnormalities in the offspring, including long-term alterations in lipid metabolism and pancreatic function [53]. Exposure to maternal hyperglycemia during fetal life has also been shown to impair glucose regulation and increase susceptibility to metabolic disease in adulthood [54].

Together, these experimental studies support the concept that fetal overgrowth represents an early manifestation of metabolic programming.

### 4.2. Human Studies on Fetal Organs: The Brain, Heart, Pancreas, Liver and Kidneys

Similar to FGR, fetal overgrowth is associated with organ-specific structural and functional adaptations, although these are primarily driven by metabolic rather than hemodynamic mechanisms.

#### 4.2.1. Brain

Fetal overgrowth, particularly in the context of maternal diabetes and metabolic dysregulation, reflects exposure to an intrauterine environment characterized by hyperglycemia and altered nutrient availability. Importantly, available evidence suggests that neurodevelopmental alterations in these offspring are driven by the intrauterine metabolic milieu rather than fetal size itself [55]. In contrast to FGR, where placental insufficiency predominates, these effects are primarily mediated by metabolic rather than hemodynamic factors.

Neuroimaging studies suggest that exposure to maternal diabetes is associated with subtle structural brain changes, including reduced cortical thickness and regional alterations in brain morphology, indicating that excess intrauterine nutrient exposure may affect cortical development [56].

In addition, region-specific structural alterations have been described in the hippocampus, a key structure involved in memory and emotional regulation, with evidence of altered morphology in children exposed to gestational diabetes [57].

Advanced imaging further demonstrates microstructural abnormalities in white matter organization in infants of mothers with gestational diabetes, including reduced fractional anisotropy in major white matter tracts, which has been associated with impaired neurocognitive performance, suggesting early disruption of brain connectivity [58].

These structural and microstructural changes translate clinically into an increased risk of neurodevelopmental impairment. Large-scale epidemiological data demonstrate that maternal diabetes is associated with higher risks of neurodevelopmental disorders, including attention-deficit/hyperactivity disorder, autism spectrum disorder, and cognitive impairment, with stronger associations observed in pregestational diabetes compared to gestational diabetes [55].

#### 4.2.2. Heart and Vascular System

Exposure to maternal diabetes and fetal overgrowth has been associated with early cardiac remodeling, including increased cardiac dimensions, myocardial hypertrophy, and alterations in cardiac geometry [59,60,61]. Functional alterations have also been described, with impaired myocardial deformation and subclinical cardiac dysfunction persisting into early childhood in the offspring of mothers with diabetes. Although left ventricular hypertrophy may partially regress after birth, the persistence of increased ventricular mass in some infants suggests the incomplete normalization of myocardial remodeling [62,63].

Beyond the myocardium, vascular alterations have been demonstrated, with increased carotid intima-media thickness observed in both overgrowth infants and the offspring of diabetic mothers during childhood, indicating early vascular remodeling and a potential predisposition to atherosclerosis [64].

At the population level, LGA birth has been associated with increased long-term cardiovascular risk, including atrial fibrillation, supporting the concept of fetal programming of cardiovascular disease in the setting of intrauterine overnutrition [65].

#### 4.2.3. Pancreas

Fetal overgrowth, particularly in the context of maternal hyperglycemia, is associated with alterations in fetal pancreatic development and insulin regulation. Maternal hyperglycemia leads to fetal hyperglycemia and consequent stimulation of pancreatic β-cells, resulting in fetal hyperinsulinemia, which is a key driver of accelerated somatic growth and adiposity [66].

Experimental and histopathological data further suggest that overgrown fetuses demonstrate β-cell hyperplasia and increased islet size, consistent with sustained intrauterine insulin stimulation, although these findings are derived mainly from small cohorts and postmortem studies [67].

Children born LGA exhibit alterations in glucose homeostasis, including higher fasting insulin levels and increased insulin resistance during childhood, even in the absence of overt maternal diabetes, suggesting early metabolic programming of pancreatic function [66].

Interestingly, data from the Hyperglycemia and Adverse Pregnancy Outcome (HAPO) Follow-Up Study demonstrate that, after adjustment for maternal glucose levels during pregnancy, higher birthweight and neonatal adiposity are associated with greater insulin sensitivity and lower glucose levels in childhood, highlighting the complex relationship between birth size and metabolic outcomes and underscoring the dominant influence of the intrauterine metabolic environment rather than birth size alone [68].

#### 4.2.4. Liver

Histopathological evidence demonstrates that fetal exposure to maternal diabetes is associated with increased hepatic fat accumulation, with a markedly higher prevalence and severity of hepatic steatosis observed in stillborns of diabetic mothers compared with controls, independent of maternal obesity and fetal overgrowth. These findings suggest that fetal hepatic lipid accumulation may represent a direct response to the diabetic intrauterine environment rather than a consequence of increased fetal size alone [69].

Consistent with this, epidemiological data indicate that offspring of pregnancies complicated by gestational diabetes have an increased risk of developing NAFLD during childhood and early adulthood, with meta-analytic evidence demonstrating approximately a two-fold increased risk compared to offspring of non-diabetic pregnancies [70].

#### 4.2.5. Kidneys

Data regarding renal development in fetal overgrowth are limited, and available evidence suggests that potential long-term renal effects are more closely related to maternal metabolic conditions, particularly diabetes, than to fetal size itself. Experimental studies indicate that exposure to maternal diabetes may affect nephrogenesis, including altered differentiation of nephron progenitor cells and a potential reduction in nephron number [71].

Prenatal exposure to maternal diabetes has been associated with subtle alterations in renal function later in life. Adult offspring of mothers with type 1 diabetes demonstrate reduced renal functional reserve, suggesting a potential vulnerability to renal stress, although baseline renal function is often preserved [72].

Population-based studies further suggest that maternal diabetes, as well as maternal overweight and obesity, are associated with an increased risk of childhood chronic kidney disease, whereas high birth weight itself does not remain independently associated after adjustment for maternal factors, supporting the concept that intrauterine metabolic exposure rather than overgrowth per se is the primary determinant of later renal risk [73]. Postnatal growth patterns appear to modify later risk, with term LGA infants without catch-down growth showing increased odds of elevated blood pressure at childhood [74].

## 5. The Placental Function and Dysfunction

Normal fetal growth depends on the adequate development and function of the placenta, which serves as the interface for oxygen and nutrient exchange between the maternal and fetal circulations [75,76]. This process requires coordinated remodeling of the uteroplacental circulation, appropriate villous development, and efficient transport across the syncytiotrophoblast [76,77].

Placental-mediated FGR most commonly arises from impaired trophoblast invasion and inadequate remodeling of the maternal spiral arteries, leading to reduced uteroplacental perfusion and chronic fetal hypoxia [78]. The resulting malperfusion induces placental oxidative stress, inflammation, and structural abnormalities, including reduced villous volume, increased infarction, and fibrin deposition, ultimately limiting the surface area available for maternal–fetal exchange [76].

Beyond hemodynamic impairment, placental dysfunction in FGR also reflects complex alterations in cellular signaling and transport capacity. Alterations in insulin and insulin-like growth factor pathways, which are key regulators of placental growth and nutrient transport, have been associated with impaired fetal growth and altered fetal metabolic profiles [79]. In parallel, inhibition of placental mTOR signaling, a central nutrient-sensing pathway linking maternal nutrient availability to fetal growth, leads to reduced amino acid transport and contributes directly to growth restriction [80]. These findings, together with broader regulatory changes described in human FGR placenta [77], support the concept that the placenta actively adapts to limited maternal resource availability by modulating fetal growth, rather than representing purely passive failure.

At the molecular level, placental hypoxia is a central driver of these processes, with upregulation of hypoxia-responsive pathways such as HIF1A and VEGFA contributing to altered angiogenesis and vascular development in FGR placenta [81]. These changes further disrupt placental vascular architecture and exacerbate functional impairment. Macroscopically and microscopically, FGR placentae are typically smaller and exhibit abnormal villous branching, impaired trophoblast proliferation and differentiation, and premature cellular senescence, all of which contribute to reduced exchange efficiency [75]. Collectively, these structural, functional, and molecular alterations define placental insufficiency as a multifactorial process underlying fetal growth restriction.

In fetal overgrowth, placental dysfunction is usually not characterized by classical uteroplacental insufficiency, but rather by maladaptive placental growth and enhanced nutrient transfer in pregnancies complicated mainly by maternal diabetes and obesity. Human studies have shown that diabetic pregnancies associated with accelerated fetal growth demonstrate increased placental glucose transport, with higher GLUT1 expression and glucose uptake in the basal membrane of the syncytiotrophoblast, supporting greater maternofetal glucose flux and fetal overgrowth [82]. In addition, placental insulin/IGF-I and mTOR signaling are activated in association with increasing maternal BMI and birth weight, and placental system amino acid transport activity is positively correlated with birth weight, suggesting that nutrient-sensing pathways actively contribute to excessive fetal growth rather than merely reflecting it [83].

Thus, placental dysfunction in fetal growth abnormalities reflects a spectrum ranging from impaired perfusion and reduced nutrient transfer in FGR, to enhanced nutrient transport and growth signaling in fetal overgrowth. However, the extent to which these mechanisms independently determine long-term outcomes remains incompletely understood.

Importantly, many of these placental and fetal adaptive processes can be assessed in vivo using ultrasound and Doppler techniques, providing a functional window into intrauterine conditions and their potential clinical implications.

## 6. The Contribution of Ultrasound and Doppler to the Prediction of Postnatal Health

### 6.1. Fetal Growth Restriction

Over the past two decades, ultrasound and Doppler studies in human FGR have evaluated multiple fetal organs, including the brain, heart, kidneys, adrenal glands, liver, and pancreas, in addition to the assessment of the placenta and the fetal circulation, which serve as a significant support to strengthen the thrifty phenotype hypothesis [4,5,11]. Neurosonography has shown delayed brain maturation in late-onset FGR, particularly in fetuses with abnormal Doppler findings, with smaller corpus callosum and cortical fissure measurements [84], while fetal echocardiography has consistently demonstrated cardiac remodeling and dysfunction in both early- and late-onset FGR, even before overt clinical deterioration [85,86,87,88,89]. These observations suggest that prenatal imaging may identify not only the growth-restricted fetus, but also the pattern of organ involvement that could underlie later postnatal morbidity.

Our group previously reported an illustrative case of early severe FGR and oligohydramnios, secondary to placental compromise, characterized by arterial redistribution and reduced resistance to blood flow in the fetal brain (brain-sparing), with consequent probable ischemic changes in other fetal organs. The kidneys and pancreas were below the 5th percentile for gestational age, accompanied by impaired cardiac function and liver porto-systemic venous shunting [11].

A recent study further suggests that ultrasound assessment of the fetal pancreas may add a metabolic dimension to FGR, as reduced pancreatic circumference has been demonstrated in growth-restricted fetuses, supporting impaired pancreatic development as a possible prenatal marker of later metabolic vulnerability [90]. In addition, second trimester organ mapping suggests that pancreatic diameter and the abdomen-to-pancreas ratio may help identify fetuses at risk for low birth weight or growth restriction, although these findings still require further validation [91].

Venous Doppler and targeted assessment of the fetal portal system may also refine the phenotypic classification of FGR. Intrahepatic umbilical-porto-systemic venous shunts have been associated with fetal growth restriction, earlier presentation, abnormal ductus venosus Doppler and increased fetal death, suggesting that diversion of umbilical flow away from the liver may contribute to a distinct and more severe subtype of FGR in some cases [92,93].

**Figure 3:**
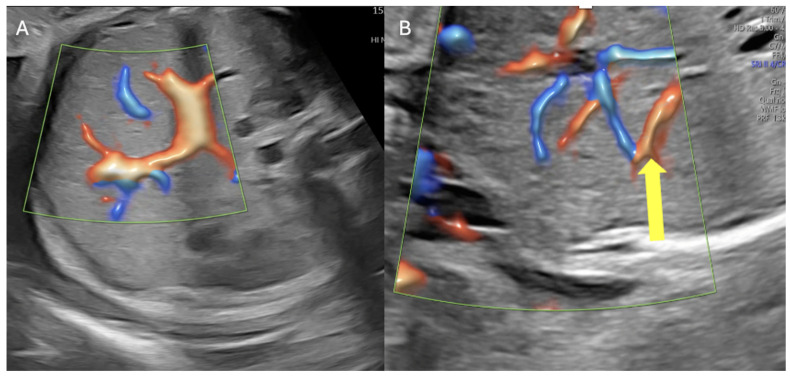
Intrauterine undernutrition: intrahepatic portosystemic shunt in fetal growth restriction. (**A**) Normal fetal portal venous anatomy. (**B**) Ultrasound image at 26 weeks’ gestation demonstrating an intrahepatic portosystemic shunt (yellow arrow) in a fetus with growth restriction, that may lead to ischemic changes to the fetal liver, who is prone to develop future complications as a result [94].

Our group has recently published a case series of severe FGR and oligohydramnios, in which arterial redistribution by liver porto-systemic venous shunting appeared to serve as a rescue mechanism to improve fetal growth and amniotic fluid amount (Figure 3); this shunting resolved during postnatal follow-up up to 30 months after birth [94].

### 6.2. Fetal Overgrowth

Ultrasound studies in fetal overgrowth, particularly in the context of maternal diabetes and metabolic dysregulation, suggest that excessive intrauterine nutrient exposure affects not only fetal size, but also fetal organ development and function.

Our group identified an association between increased fetal pancreatic size and the subsequent development of gestational diabetes [95].

**Figure 4:**
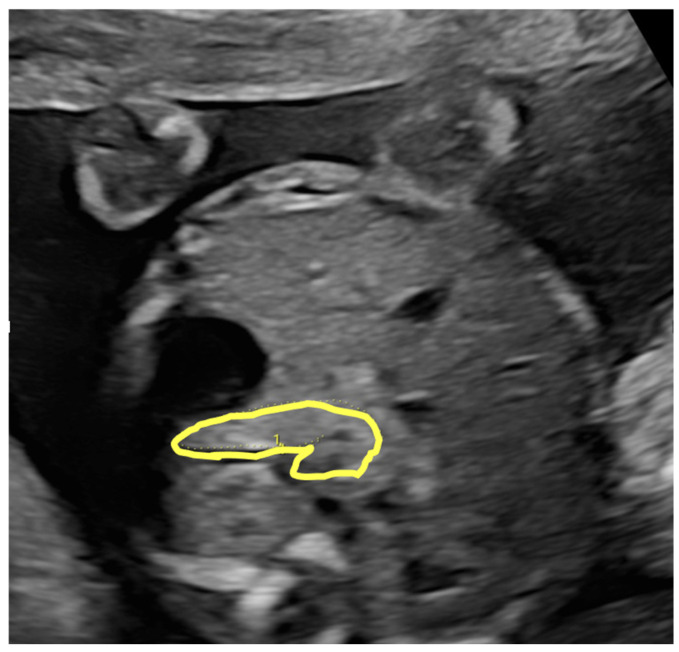
Intrauterine overnutrition: enlarged fetal pancreas preceding gestational diabetes diagnosis. Ultrasound at 22 + 0 weeks demonstrating enlarged fetal pancreas (>90th percentile), with the pancreatic circumference highlighted in yellow, preceding subsequent diagnosis of gestational diabetes mellitus. This fetus with an enlarged pancreas is prone to develop future impaired glucose tolerance.

These intrauterine conditions may contribute to childhood obesity, IGT, and later metabolic syndrome, possibly related to the effects of fetal pancreatic enlargement during intrauterine life [6,10]. In support of this, fetal pancreatic size was shown to correlate with maternal glycemic status, fetal weight and gestational age, reflecting fetal hyperinsulinemia and suggesting that pancreatic hypertrophy represents an early marker of metabolic programming in utero (Figure 4) [96].

Fetal echocardiographic studies further demonstrate that maternal diabetes is associated with cardiac remodeling and functional alterations, including myocardial hypertrophy, impaired diastolic function and reduced myocardial performance, even in the absence of structural heart disease [97]. These changes may be detected already in mid-gestation, prior to the clinical diagnosis of gestational diabetes, suggesting that maternal metabolic risk factors contribute to early fetal cardiac programming [98]. In addition, fetuses exposed to gestational diabetes demonstrate subtle reductions in right ventricular function and altered cardiac geometry, indicating early myocardial adaptation to the intrauterine metabolic environment [99].

Importantly, fetal echocardiography in the third trimester has shown that fetuses with overgrowth, subsequently born as LGA newborns, may present with functional cardiovascular abnormalities, including ductal constriction, myocardial hypertrophy, and cardiomegaly, identified more frequently compared with appropriately grown fetuses, suggesting that ultrasound can detect early functional cardiac differences in the context of fetal overgrowth [100].

## 7. Conclusions

In summary, ultrasound and Doppler assessment provide a unique opportunity to evaluate the intrauterine environment beyond fetal size alone, by demonstrating organ-specific structural and functional adaptations in both undernutrition and overnutrition. These findings may offer insight into early pathways linking fetal conditions with later health, although further longitudinal studies are required to better define these associations.

From a clinical perspective, these findings may have implications already in the neonatal period. Prenatal Doppler and organ-specific imaging findings could contribute to the early identification of newborns at increased risk, potentially guiding targeted neonatal monitoring, including metabolic screening, cardiovascular evaluation, and neurodevelopmental follow-up. Integration of prenatal imaging with postnatal risk stratification may represent an important step toward more personalized, longitudinal care.

## Data Availability

No new data were created or analyzed in this study. Data sharing is not applicable to this article.

## Abbreviations

The following abbreviations are used in this manuscript:

FGR	Fetal growth restriction
SGA	Small for gestational age
LBW	Low birth weight
LGA	Large for gestational age
IGT	Impaired glucose tolerance
HAPO	Hyperglycemia and adverse pregnancy outcome
NAFLD	Non-alcoholic fatty liver disease
MCA	Middle cerebral artery
